# Dural venous sinus anatomy in children with external hydrocephalus: analysis of a series of 97 patients

**DOI:** 10.1007/s00381-021-05322-5

**Published:** 2021-08-24

**Authors:** Giuseppe Cinalli, Giuliana di Martino, Carmela Russo, Federica Mazio, Anna Nastro, Giuseppe Mirone, Claudio Ruggiero, Ferdinando Aliberti, Daniele Cascone, Eugenio Covelli, Pietro Spennato

**Affiliations:** 1 Department of Pediatric Neurosciences, Pediatric Neurosurgery Unit, Santobono-Pausilipon Children’s Hospital, Via Mario Fiore n. 6, 80129 Naples, Italy; 2 Department of Pediatric Neurosciences, Pediatric Neuroradiology Unit, Santobono-Pausilipon Children’s Hospital, Via Mario Fiore n. 6, 80129 Naples, Italy

**Keywords:** External hydrocephalus, Macrocrania, Children, Venous hypertension, Positional plagiocephaly, Transverse sinus, Sigmoid sinus, Venous obstruction grading score

## Abstract

**Purpose:**

To evaluate the anatomical variations of dural venous sinuses in children with external hydrocephalus, proposing a radiological grading of progressive anatomic restriction to venous outflow based on brain phase-contrast magnetic resonance venography (PC-MRV); to evaluate the correlation between positional plagiocephaly and dural sinuses patency; and to compare these findings with a control group to ascertain the role of anatomical restriction to venous outflow in the pathophysiology of external hydrocephalus.

**Methods:**

Brain MRI and PC MRV were performed in 97 children (76 males, 21 females) diagnosed with external hydrocephalus at an average age of 8.22 months. Reduction of patency of the dural sinuses was graded as 1 (stenosis), 2 (complete stop) and 3 (complete agenesis) for each transverse/sigmoid sinus and sagittal sinus. Anatomical restriction was graded for each patient from 0 (symmetric anatomy of patent dural sinuses) through 6 (bilateral agenesis of both transverse sinuses). Ventricular and subarachnoid spaces were measured above the intercommissural plane using segmentation software. Positional plagiocephaly (PP) and/or asymmetric tentorial insertion (ATI) was correlated with the presence and grading of venous sinus obstruction. These results were compared with a retrospective control group of 75 patients (35 males, 40 females).

**Results:**

Both the rate (84.53% vs 25.33%) and the grading (mean 2.59 vs mean 0.45) of anomalies of dural sinuses were significantly higher in case group than in control group. In the case group, sinus anomalies were asymmetric in 59 cases (right-left ratio 1/1) and symmetric in 22. A significant association was detected between the grading of venous drainage alterations and diagnosis of disease and between the severity of vascular anomalies and the widening of subarachnoid space (SAS). Postural plagiocephaly (39.1% vs 21.3%) and asymmetric tentorial insertion (35.4% vs 17.3%) were significantly more frequent in the case group than in the control group. When sinus anomalies occurred in plagiocephalic children, the obstruction grading was significantly higher on the flattened side (*p* ≤ 0.001).

**Conclusion:**

Decreased patency of the dural sinuses and consequent increased venous outflow resistance may play a role in the pathophysiology of external hydrocephalus in the first 3 years of life. In plagiocephalic children, calvarial flattening may impact on the homolateral dural sinus patency, with a possible effect on the anatomy of dural sinuses and venous drainage in the first months of life.

**Supplementary information:**

The online version contains supplementary material available at 10.1007/s00381-021-05322-5.

## Introduction

External hydrocephalus, also known as subarachnomegaly, benign pericerebral collections or benign enlargement of the subarachnoid spaces (BESS), is a childhood condition, characterized by macrocrania and widening of the subarachnoid spaces, especially in the frontal area, frequently associated with mild ventricular dilatation and with generally benign and self-limiting course [1–12]. Its incidence is not very well known but has been recently estimated at 0.4 per 1000 live births in a population-based study, accounting for approximately half that of all primary hydrocephalus (0.75 per 1000 live births) in a pediatric setting in the same population [11]. In most cases, it is idiopathic; secondary forms may be related to prematurity, intraventricular hemorrhage, subdural hematoma, meningitis, or increased intracranial venous pressure [3, 4, 6, 11, 12]. In this paper, we prefer the term “external hydrocephalus” because we do not like to use the term “benign” for a condition that has been frequently associated with some degree of early delayed development; can have, although rarely, surgical implications; and finally, it is not limited to subarachnoid or pericerebral spaces since significant ventriculomegaly is a constant part of the neuroradiological features. Actually, there is a gap of knowledge about the underlying pathophysiology that remains largely unclear. Classic Barlow’s theory (immaturity of arachnoid granulations) is not applicable in all cases, particularly in early infantile period [13].

The venous system represents the main CSF-outflow pathway both in “major” (via arachnoid villi) and in “minor” (via extra-arachnoid villi sites) CSF pathways [14]. The transverse-sigmoid sinuses work both as superficial and deep drainage system, and their morphology has been hypothesized to be influenced by development of the brain and postural hemodynamic changes [15]. However, the impact of primary intracranial venous hypertension (VHT) on pathophysiology of the CSF dynamics in external hydrocephalus is still little researched and probably underestimated. The aim of this study is to investigate the prevalence and possible role of the altered intracranial venous drainage in external hydrocephalus through a retrospective case–control study and a review of the literature.

## Methods

### Patient group

The clinical records, neuroimaging, surgical findings, and clinical follow-up of ninety-seven patients with diagnosis of macrocrania and widened subarachnoid spaces admitted to our department between 2006 and 2020 were retrospectively reviewed. Inclusion criteria were age below 26 months at time of diagnosis, head circumference greater than 95th percentile on the standard growth curve in the first year of life, typical enlargement of subarachnoid spaces documented by neuroimaging, and at least one venographic study with brain magnetic resonance phase-contrast venography (PC MRV) in the diagnostic work up. Exclusion criteria were craniosynostosis, achondroplasia, and unavailability of PC MRV sequences.

### Control group

In our department, PC MRV sequences are not part of the panel of basal brain MR sequences performed and are included in the sequence list by the neuroradiologist according with the original clinical requests. For this reason, radiological archives of the years 2007–2020 were retrospectively searched for children matching inclusion criteria for age and available PC MRV sequences acquired with the same technique as case group. Macrocrania, achondroplasia, hydrocephalus, subdural hygromas, posterior fossa tumors, vascular malformations, and craniosynostosis were considered as exclusion criteria.

### Neuroimaging

Neuroradiological diagnosis of the pericerebral collections was firstly obtained by either ultrasound, computed tomography (CT) scan, or MRI. In all included cases, brain MRI was performed on a 1.5 T scanner (Philips Medical Systems AchievaDStream, Best, The Netherlands) in supine position, with pharmacological sedation and without gadolinium administration. Standardized protocol of brain MRI consisted of axial, sagittal, and coronal fast spin-echo T2-weighted imaging, axial spin-echo T1-weighted imaging. Other sequences were performed as clinically indicated. Images were systematically and independently reviewed by two pediatric neuroradiologists and two pediatric neurosurgeons.

Intracranial volume, subarachnoid space (SAS) volume, and ventricular volumes were calculated per each patient by segmentation software (HOROS Project, GNU Lesser General Public License, Version 3.0 (LGPL 3.0)) on axial T2-weighted images 2 mm thick. Brush ROI method was used for segmentation of subarachnoid spaces and pencil ROI for intracranial volume and ventricular volume segmentation.

The entity of widening of subarachnoid spaces was evaluated measuring the amount of cerebrospinal fluid of the convexity from the intercommissural plane until the last cut of the vertex where SAS were visible [16]. Accordingly, ventricular volumes were calculated measuring the volume of the third and lateral ventricle from intercommissural plane to vertex. In addition, CSF volumes were normalized with intracranial volume, and they were grouped according to the grading of alterations of intracranial venous drainage. The presence of right or left positional plagiocephaly was recorded. The presence of significant asymmetry of insertion of the falx on the tentorium (asymmetric tentorial insertion = ATI) was recorded as anatomical variant possibly impacting the anatomy of dural venous sinuses in the region of the torcular herophili.

**Fig. 1:**
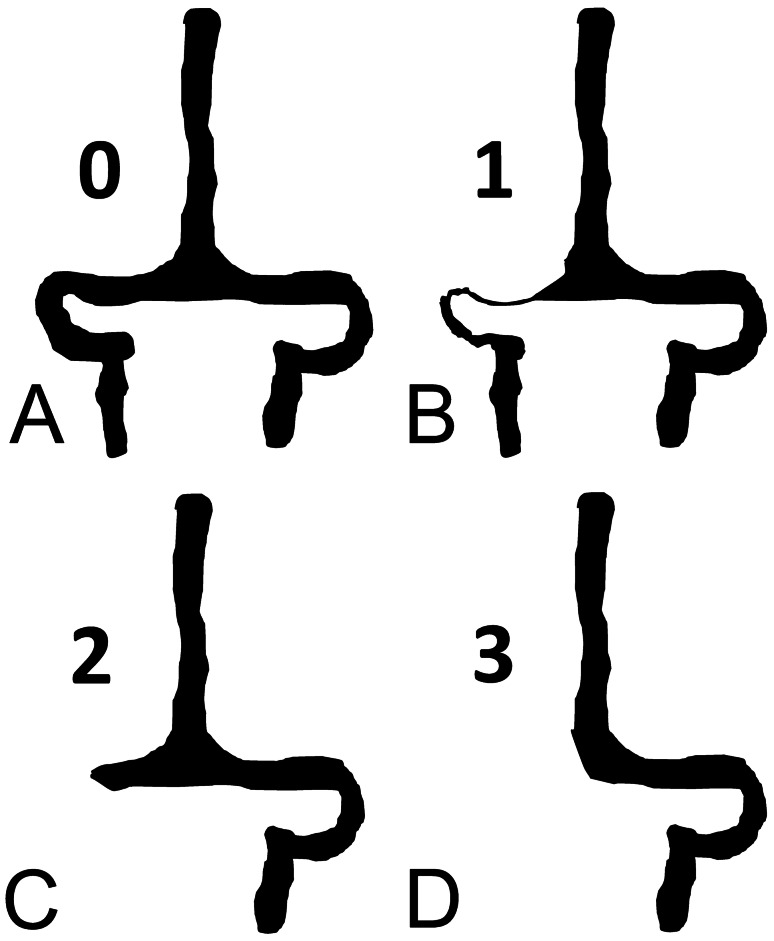
Schematic drawing of venous obstruction grading score. **A** Grade 0: transverse and sigmoid sinus are well visible from the torcular herophili to the jugular foramen in full continuity with Jugular vein without significant variations in diameter throughout the length. **B** Grade 1: a significant reduction of diameter (> 50%) is well visible. **C** Grade 2: a complete gap of flow signal is visible. **D** Grade 3: complete aplasia of the lateral sinus. A grading is assigned to each side (right + left) and the two gradings are added together (Complete detail of the grading system with several radiological MRV examples are available in Supplementary Material available in the online version at: 10.1007/s00381-021-05322-5)

MRV was performed by using phase-contrast (PC) MRV in the three standard spatial orientations (TR, 27 ms; TE, 12 ms; flip angle, 20 degrees; section thickness, 1.53 mm; overlap, 1.5; number of signals, 1; number of partition, 95; VENC factor for image acquisition, 15 cm/s). Post-processing of PC MRV images for 3D reconstructions was performed by using the maximum intensity projection (MIP) and creating 12 MIP projections at 13° intervals. Venogram assessment included superior sagittal sinus, torcular herophili, straight sinus, transverse and sigmoid sinuses, jugular bulbs, occipital sinuses, and major intracranial veins (internal cerebral veins, basal vein of Rosenthal, vein of Galen, vein of Trolard, vein of Labbè, and internal jugular veins). Collateral circle was also evaluated. In particular, for sagittal sinus and each transverse-sigmoid-jugular venous territory, we researched the presence of stenosis (scored 1), flow gap (scored 2), and agenesis (scored 3) (Fig. 1). Bilateral alterations were summed up. A grading classification was therefore created by assigning a score: grade 0, no visible narrowing of sagittal, transverse or sigmoid sinuses (Supplementary Fig. 1); grade 1, stenosis of one lateral or sigmoid dural sinus (Supplementary Fig. 2); grade 2, bilateral stenosis of two dural sinuses (grade 2A) (Supplementary Fig. 3) or flow gap in one dural sinus (grade 2B) (Supplementary Fig. 4) or focal stenosis of the sagittal sinus (Grade 2C) (Supplementary Fig. 5); grade 3, flow gap on one side plus contralateral stenosis (grade 3A) (Supplementary Fig. 6) or aplasia of one dural sinus (grade 3B) (Supplementary Fig. 7); grade 4, bilateral flow gap (grade 4A) (Supplementary Fig. 8) or aplasia of a dural sinus plus contralateral stenosis or (grade 4B) (Supplementary Fig. 9); grade 5, aplasia of a dural sinus plus contralateral flow gap (Supplementary Fig. 10); grade 6, bilateral aplasia of both dural sinuses (Supplementary Fig. 11). The final grade was assigned after thorough evaluation by two separate neuroradiologists and two separate neurosurgeons of all anatomical projections (lateral, vertical, anteroposterior) of the 3D reconstruction of the dural venous system in the region of torcular herophili. In case of disagreement, the final score was assigned in collegial discussion after revision of the images.

### Statistical analysis

All variables were previously checked for missing data and outliers. The Wilcoxon signed-rank test was used to assess the differences in clinical and neuroradiological data between patients and control group. In detail, SAS volumes, ventricular volumes, intracranial volumes, positional plagiocephaly, and asymmetric tentorial insertion (ATI) were compared between cases and control group. The Spearman’s rank correlation coefficient (ρ), corrected for ties, was used to evaluate the correlation between the grading of venous drainage abnormalities and the diagnosis of CSF collections and between the grading of venous drainage abnormalities and SAS and ventricular volume in the patients’ group, both in absolute and normalized values. All *P*-values were based on two-tailed test, with a statistical significance set at *P* ≤ 0.05.

## Results

### Patient group

**Fig. 2:**
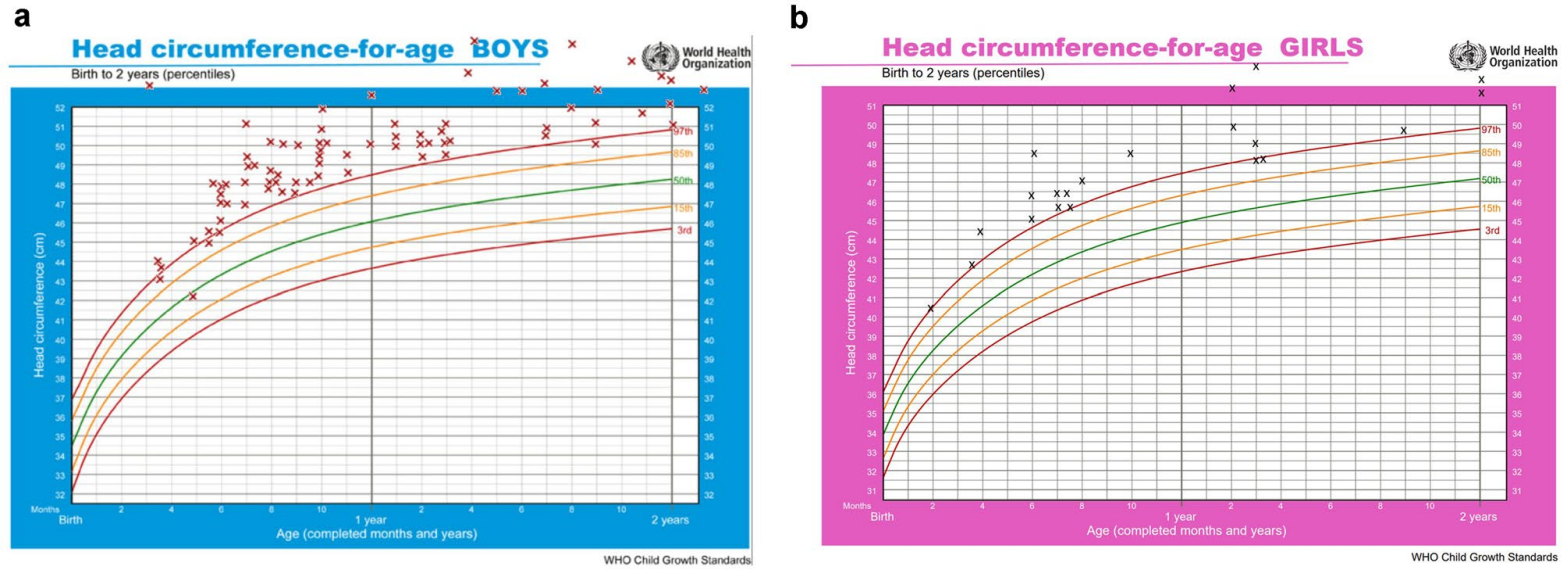
Distribution of head circumference measures of 76 male (**A**) and 21 female controls (**B**). All patients were above 95th percentile. The only patients apparently below the 95th percentile were in fact a premature well above the 99th percentile according with head growth charts modified for prematurity

Among 119 patients with diagnosis of external hydrocephalus, admitted to our department between 2006 and 2020, we excluded 22 cases who did not meet the inclusion criteria. The 97 patients remaining (76 boys and 21 girls) ranged in age at diagnosis from 2 to 26 months (mean age 8.24 months). Mean head circumference was 99.18 percentile (median 99.8) (Fig. 2A, B). No complications were reported during brain MRI with 2D MRV. Subarachnoid spaces showed typical widening in all cases, with Odita’s grade I in 57 (58.76%) cases and grade II in 40 (41.23%) cases. In 4 cases, concomitant minor subdural effusions were observed. Associated conditions were prematurity (3 cases), perinatal hypoxia (1 case), Chiari I malformation (2 cases), hyperthelorism (1 case), and syndactyly (1 case). Development was apparently normal in all patients except for 1 child (1.39%) in which psychomotor delay was observed. Signs and symptoms of intracranial hypertension occurred in 6 cases, and they underwent shunt placement (4 subduroperitoneal and 1 lumboperitoneal shunts, 1 external ventricular drainage). Mean age at PC MRV was 15.04 months.

Relevant alterations of intracranial venous drainage were observed in 82 (84.53%) patients (Table 1). Flow abnormalities were found in dural venous sinuses of the posterior fossa (80 patients) and/or in superior sagittal sinus (2 patients). They consisted in grade 1 alterations in 16/97 (16.49%) cases, grade 2 in 14/97 (14.43%) cases (6 grade 2A, 6 grade 2B, 2 grade 2C), grade 3 in 18/97 (18.55%) cases (15 grade 3A, 3 grade 3B), grade 4 in 25/97 (25.77%) cases (11 grade 4A, 14 grade 4C), grade 5 in 8/97 (8.24%) cases, and grade 6 in 1/97 (1.03%) case. Asymmetry in venous anomalies was observed in 60 cases; in 34, the predominant obstruction was on the right transverse-sigmoido-jugular axis, and in 26 on the left side.

**Table 1:** Main clinical and neuroradiological features of patients and control population

**Characteristics**	**Patients group**	**Control group**	***P*-value**
**Number of children**	97	75	
**Gender**			
Male	76 (75.35%)	35 (46.66%)	< 0.001
Female	21 (21.64%)	40 (53.33%)	
**Age at diagnosis (months)**			
Mean	8.24	NA	
Median	7	NA	
Range	1 to 26	NA	
Standard deviation	4.81	NA	
**Head circumference (percentiles)**			
Mean	99.18	44.77	< 0.001
Median	99.8	46	< 0.001
**Age at first MRV (months)**			
Mean	13.98	14.65	0.94
Median	11	11	
Range	4 to 46	1 to 47	
Standard deviation	8.78	10.58	
**Odita's grading**			
Grade I	57 (58.76%)		
Grade II	40 (41.23%)		
**Alterations of intracranial venous drainage**	82 (84.53%)	19 (25.33%)	< 0.001
Grade 1	16/97 (16.49%)	10/75 (13.3%)	< 0.001
Grade 2	14/97 (14.43%)	5/75 (6.66%)	
Grade 3	18/97 (18.55%)	3/75 (4%)	
Grade 4	25/97 (25.77%)	–	
Grade 5	8/97 (8.24%)	1/75 (1.3)	
Grade 6	1/97 (1.03%)	–	

**Fig. 3:**
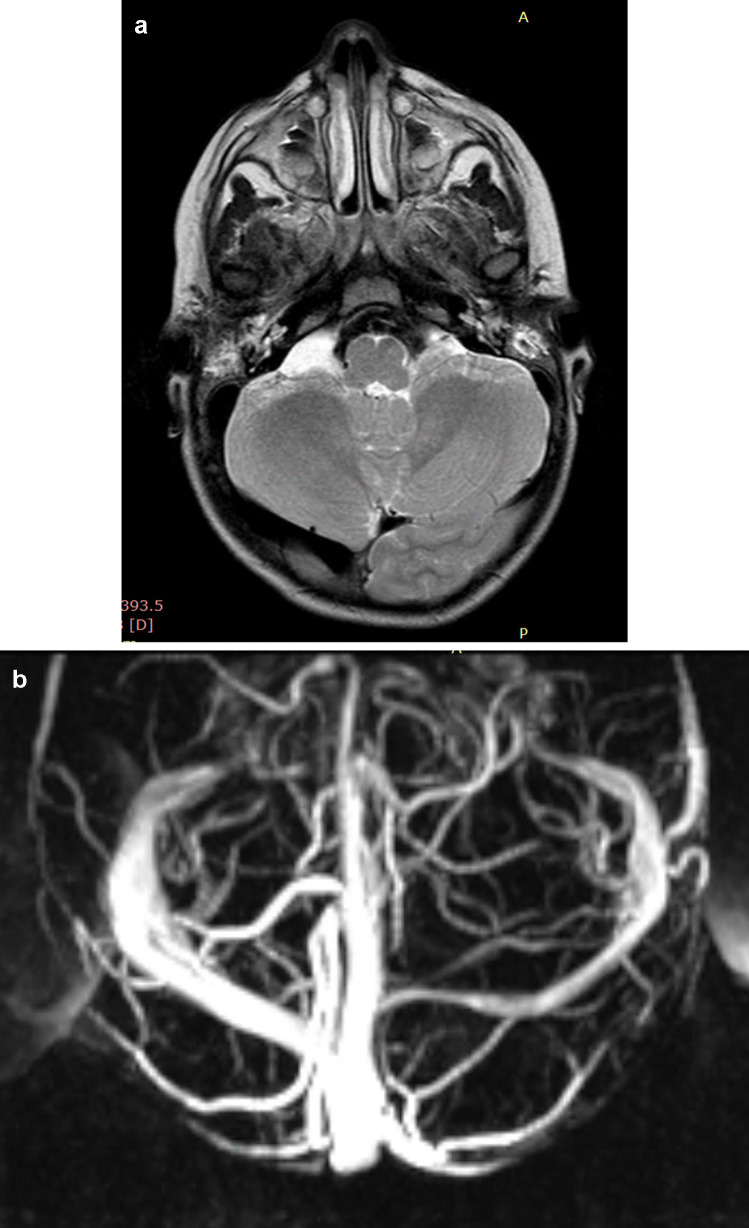
Asymmetric tentorial insertion (ATI). **A** Note the left occipital lobe crossing the midline, lying lower than the contralateral lobe, associated with contralateral displacement of the torcular herophili. **B** Same case of (**A**); angio MRV showed asymmetric orientation of transverse sinuses, with stenosis and lower displacement of the proximal third of the left sinus

**Fig. 4:**
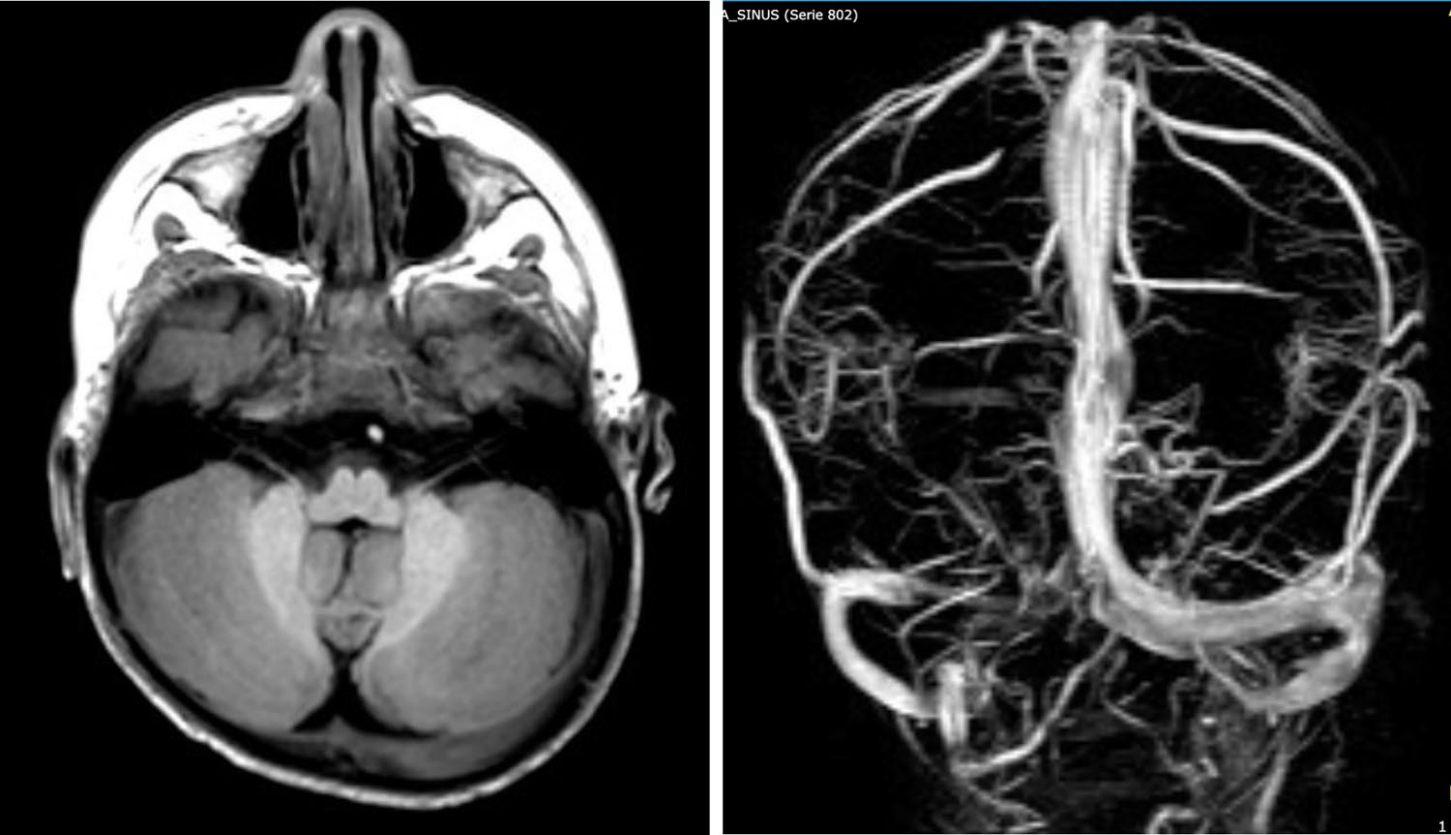
Right positional plagiocephaly. **A** Flattening of the right occipital calvaria associated with significant asymmetry of transverse sinus due to significant right hypoplasia, confirmed at angio MRV **B**

Right postural plagiocephaly was present in 21 cases, left postural plagiocephaly in 17 cases. Asymmetric tentorial insertion was observed in 34 cases (Fig. 3). Thirteen patients presented both anatomical variants. In asymmetric dural sinus anomalies, the most severe alterations were significantly associated with the flattened side of patients with postural plagiocephaly, both on the right side (*p* ≤ 0.001) and on the left side (*p* ≤ 0.001) (Fig. 4).

**Fig. 5:**
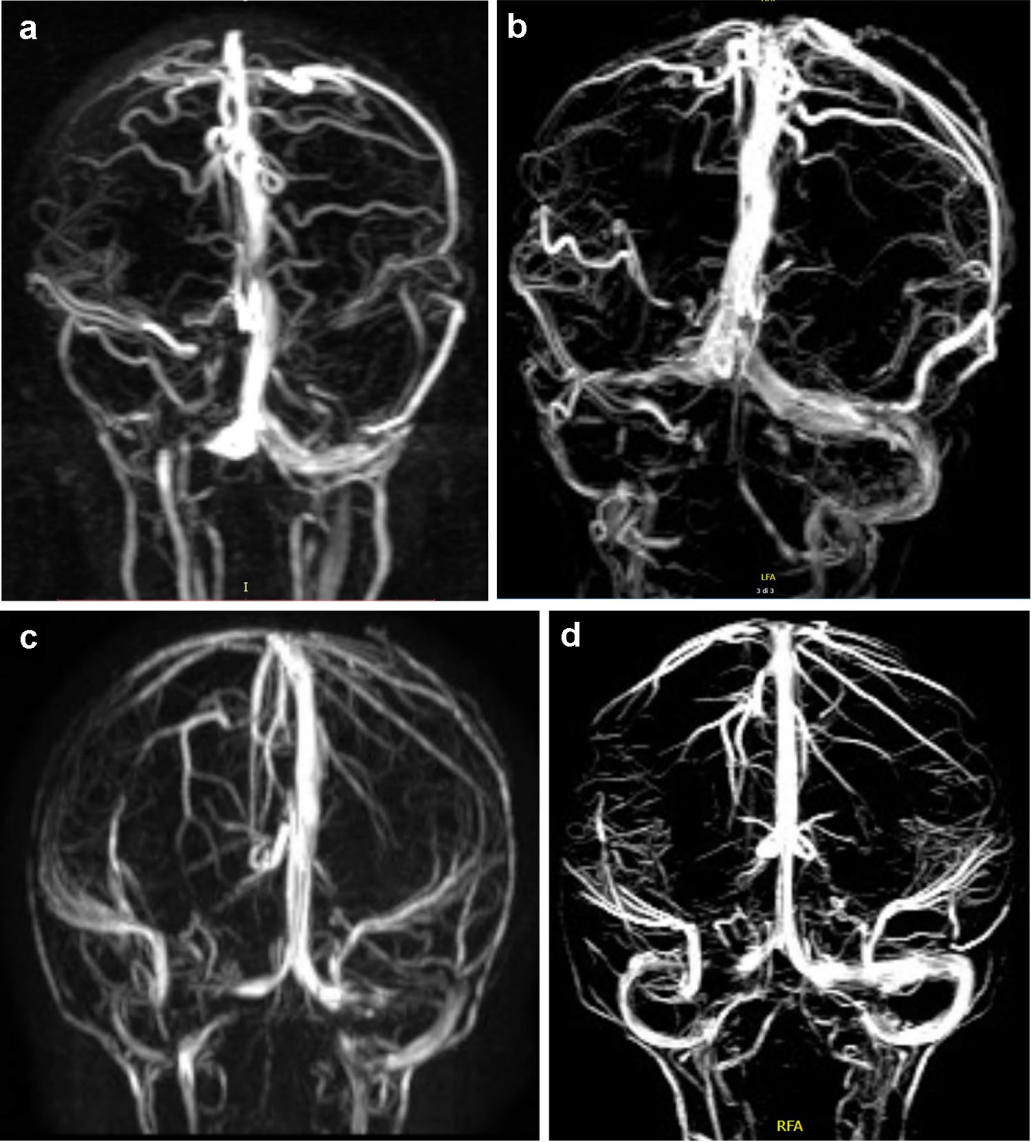
Modifications of dural venous anatomy and grading with age. **A** Eleven-month-old baby girl presenting with M/WSS. Angio MRV shows sharp flow gap at the level of the proximal third of the right transvers sinus (grading 2B). **B** Same patient at the age of 6 years and 6 months: angio MRV shows patency of the right transverse sinus that appears hypoplasic but with uniform diameter (Grading 0). **C** Nine-month-old baby boy presenting with M/WSS. Angio MRV shows bilateral flow gap in both transverse sinuses (grading 4A). **D** Thirteen months after (**C**), flow gap is still evident in the right transverse sinus but both size and flow are improved in the left (grading 2B)

Eighteen patients underwent more than one MRV during their clinical observation. Mean interval between the first and the last MRV was 35.6 months (range 5–132 months). In 7 cases, venous grading remained the same as MRV performed at diagnosis; in 5 cases, the obstruction/stenosis improved (−1 in 3 cases, −2 in 2 cases, −3 in 1 case); in 6 cases, the grading worsened (1 point in 4 cases, 2 points in 2 cases) (Fig. 5).

### Control group

After careful evaluation of all MRI exams including MRV sequences and exclusion of all exams performed in patients not meeting the inclusion criteria, 75 patients were selected to form the control group. Forty were females, 35 males. Mean head circumference was 44.77 percentile (median 46). Reasons for performing MRI exam were cerebral malformations not involving the posterior fossa in 11 cases, maxillofacial lesions in 9 cases, developmental delay in 7 cases, suspicion of anoxic/hypoxic injury in 9 cases, seizures in 5 cases, suspicion of intracerebral hemorrhage in 3 cases, skull vault lesions in 3 cases, follow up of small (< 1 cm) intracerebral lesions in 4 cases, head trauma in 2 cases, and miscellaneous in 22 cases. As already stated, MRV sequences were performed by the neuroradiologist in addition to the basal sequences according with original clinical request. Mean age at MRV was 14.65 months (range 1–36).

In the control group, standard MRI imaging showed minor or mild parenchymal anomalies confined to the supratentorial compartment and not impacting the venous drainage in 25 cases. Positional plagiocephaly was present in 8 cases on the right side and in 8 cases on the left side. ATI was observed in 13 cases. Four patients presented both anatomical variants.

On MRV studies, relevant alterations of intracranial venous drainage were observed in 19 cases (25.33%). In all but one sinus anomalies were asymmetric. Ten of them had grade 1 alterations, five patients had grade 2 alterations, three patients had grade 3 alterations, and one patient had grade 5 alterations. No significant differences in venous drainage alterations were observed between the subgroup of 25 patients presenting supratentorial anomalies and the 50 patients with normal brain MRI.

### Patient group versus control group

**Fig. 6:**
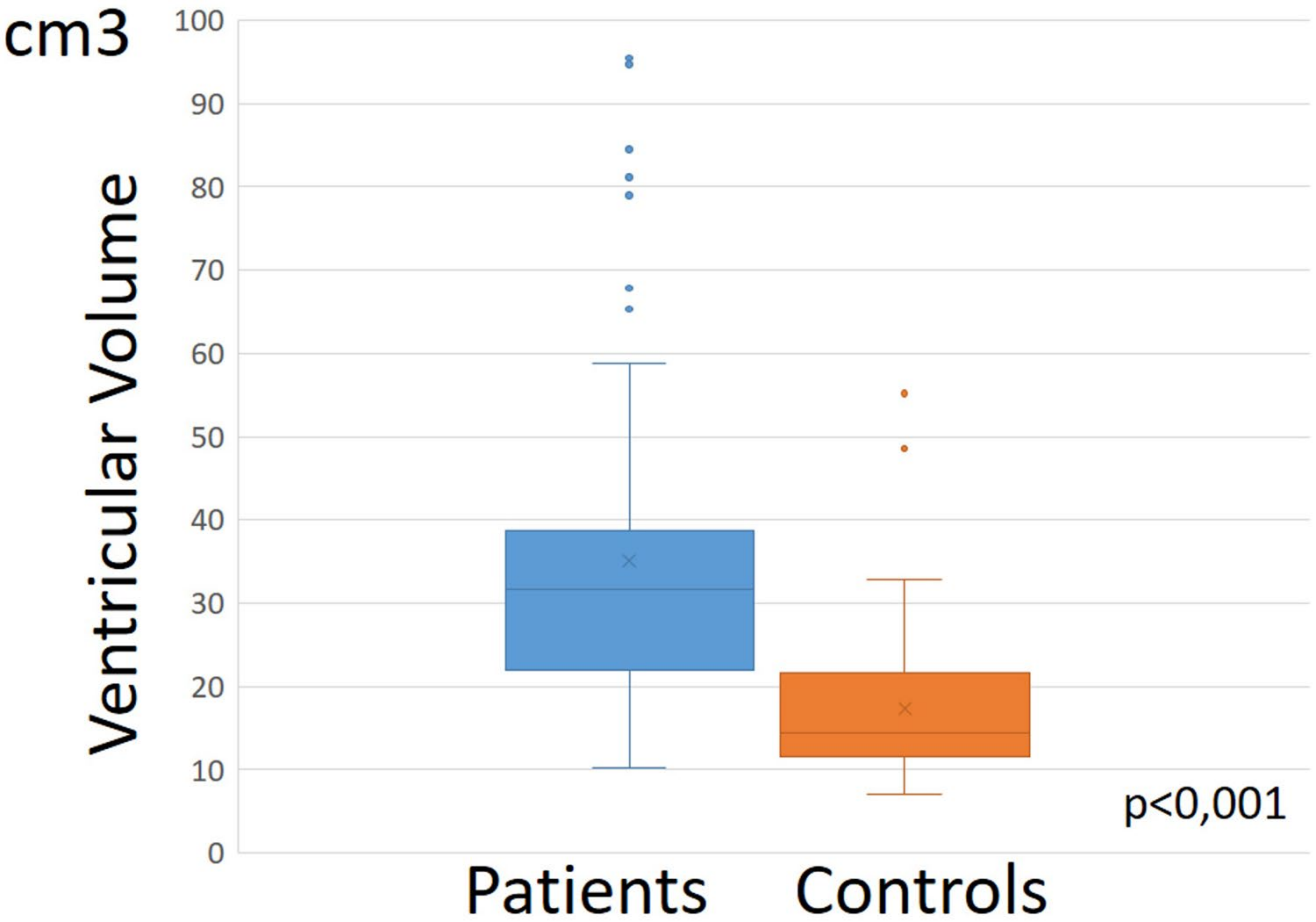
Ventricular volumes in case group were significantly larger than in control group

**Fig. 7:**
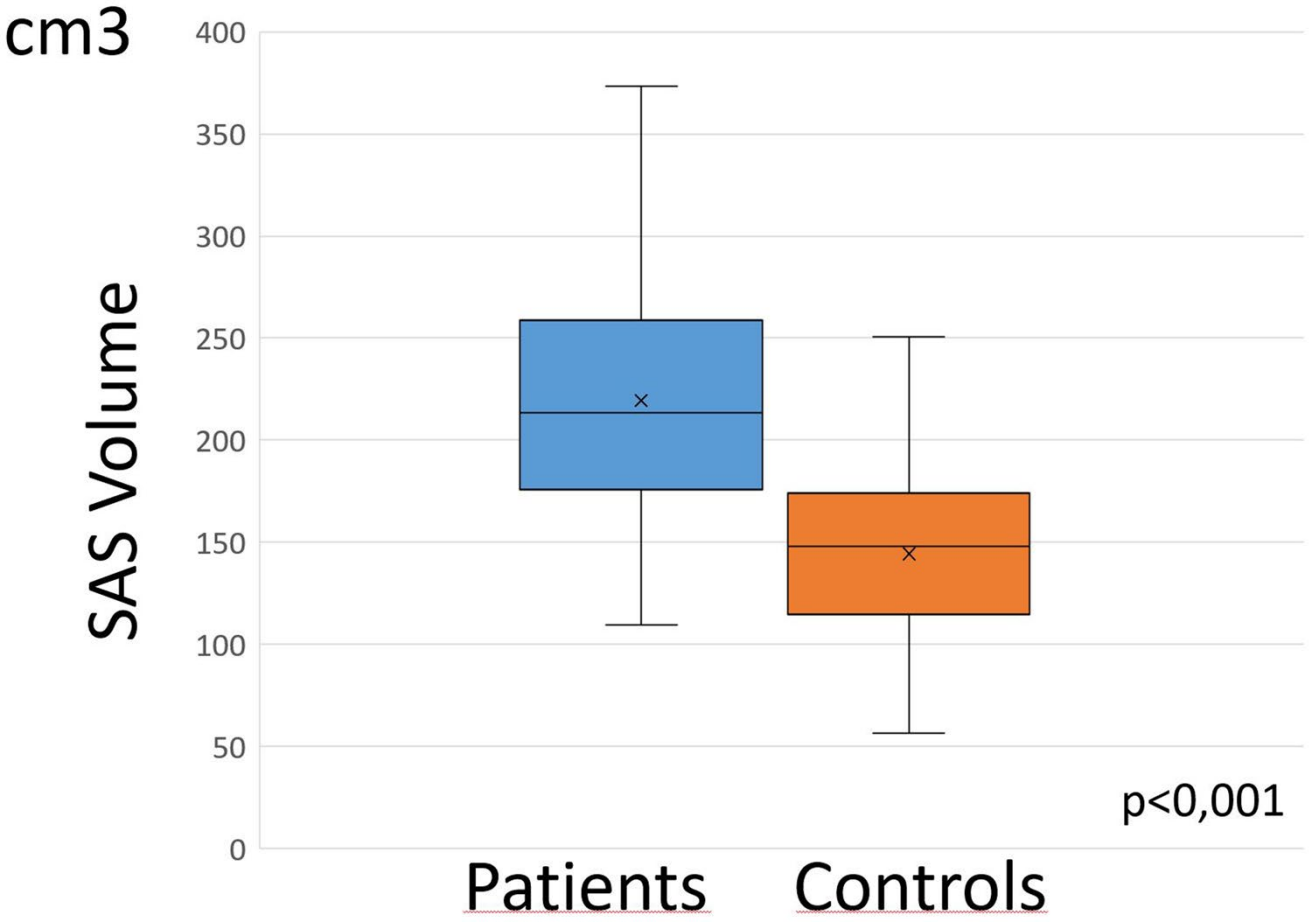
Subarachnoid spaces volume of the convexities in case group were significantly larger than in control group

By definition, mean head circumference was significantly greater in the case group (99.18 vs 44.77 percentile, *p* < 0.001). As expected, the case group had significantly larger intracranial volumes (*p* ≤ 0.001), larger ventricular volumes (*p* ≤ 0.001), and larger subarachnoid fluid volumes (*p* ≤ 0.001) than the control group (Figs. 6 and 7). After normalization for intracranial volumes, ventricular volumes and subarachnoid spaces of the case group remained significantly larger than the controls although to a lesser degree.

The rate of abnormal intracranial venous pathways was significantly higher in the case group than in the control group (84.53% vs 25.33%, *p* < 0.001). Also, the grading of anomalies was significantly higher than the control group (median of grade 3 versus median of grade 0, *p* < 0.001). A significant correlation (Spearman ρ corrected for ties: 0.59, *p* < 0.001) was found between the presence of venous drainage alterations and diagnosis of external hydrocephalus.

**Fig. 8:**
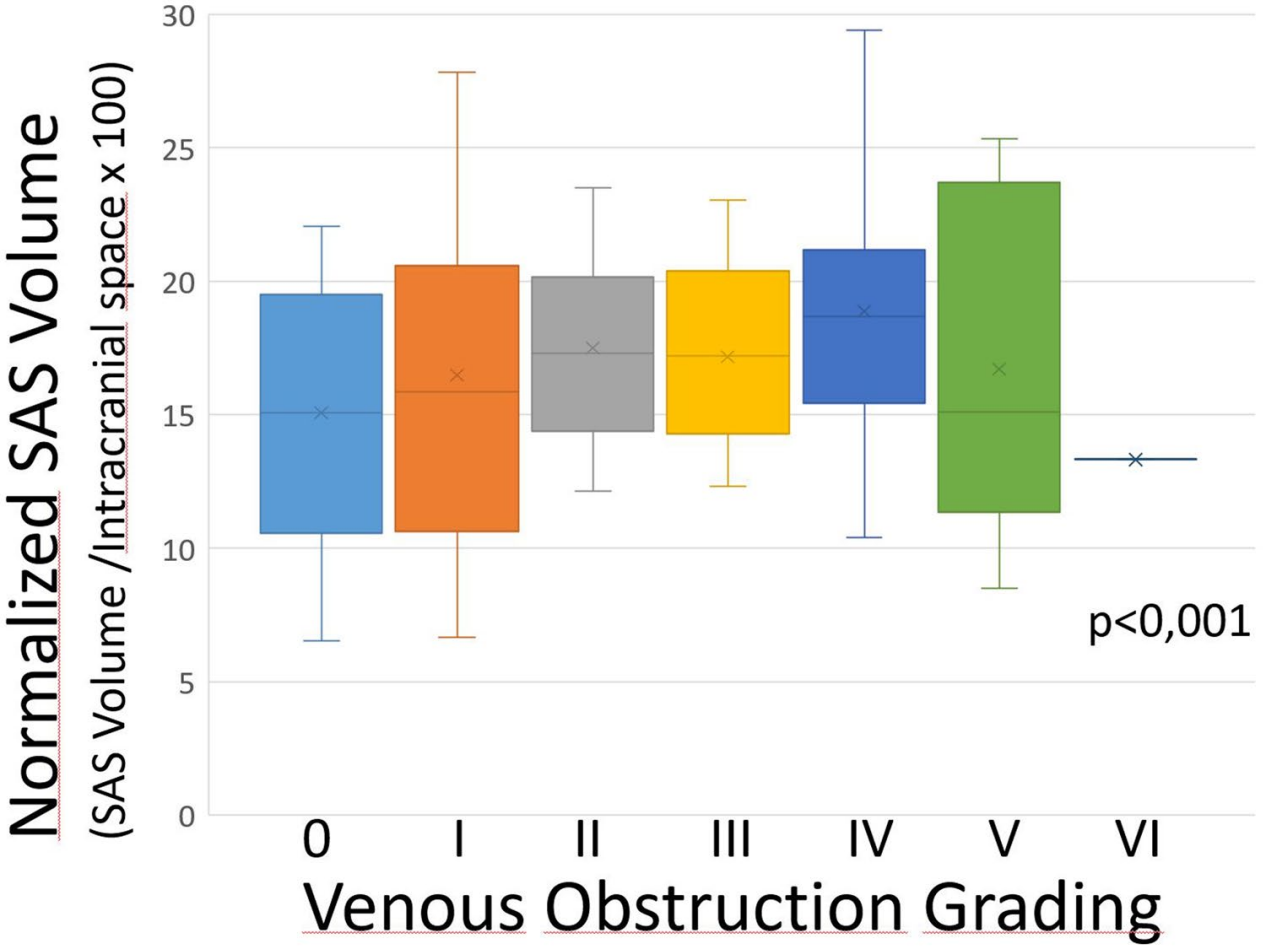
Higher venous obstruction grading was significantly correlated with larger subarachnoid spaces volume

No correlation was found between the grading of vascular anomalies and, respectively, intracranial volume (rho =  −0.22, *p* < 0.05), ventricular volume (rho =  −0.11, *p* ≥ 0.05), and subarachnoid spaces volume (Rho 0.10, *p* > 0.05). After normalization for intracranial volume (VX/intracranial volume × 100), correlation remained not significant for ventricular volumes (rho =  −0.02, *p* ≥ 0.05) whereas significant correlation was found in the case group between the grading of vascular anomalies and subarachnoid space volume (rho = 0.24, *p* = 0.01). (Fig. 8).

Postural plagiocephaly overall was significantly (*p* = 0.004) more frequent in the case group (39.17%) than in the control group (21.33%). Asymmetric tentorial insertion was significantly (*p* = 0.0096) more frequent in the case group (35%) than in the control group (17.33%), but, somehow surprisingly, this anatomical variant had no significant impact on the symmetry of dural sinus anatomy (*p* > 0.05).

## Discussion

Several authors described already in the pre-CT scan era the condition associating progressive head enlargement in the first 12 months of life without associated symptoms or other signs of intracranial hypertension, with ventricles more often of normal size or only mildly dilated, eventually associated with familial macrocephaly. Early reports were necessarily confusing and misleading, grouping these patients with others affected by various and different pathologies [17]. The condition was usually described as associated with normal psychomotor development and self-limiting evolution within 18–24 months of age [17]. Portnoy and Croissant in 1978 focused more precisely on a group of 7 normally developing male patients, presenting the clinical feature of progressive macrocephaly exceeding 2 standard deviations in the first year of life with normal ventricles or mild non-progressive ventricular dilatation, not requiring surgical treatment in 6/7 cases. Although radiological exams at that time could not display the anatomy of subarachnoid spaces, the authors performed infusion tests that revealed stagnation of radioisotope in the subarachnoid spaces over cerebral convexities. They also demonstrated in these patients normal CSF absorption rates and were able to calculate indirectly increase in sagittal sinus venous pressure in all patients, therefore associating for the first time this condition to an increased venous pressure [9], with a pathophysiological mechanism similar to that observed in other conditions like achondroplasia and craniosynostosis [18]. Sahar was the first author who clearly identified the association between macrocrania and subarachnoid space dilatation with absent or minimal ventricular dilatation [10]. With the advent of CT scan, external hydrocephalus became more and more recognized [2, 5, 7, 8, 13, 19, 20].

The development of advanced MRV techniques without gadolinium allowed easier and reliable study of intracranial venous anatomy and more precise assessment of venous anatomy alterations in the region of the torcular herophili. Short time of image acquisition, no need of contrast agent infusion, and no ionizing radiation are the main advantages [21–24]. On the contrary, less accuracy and more potential pitfalls exist in comparison with 3D MRV with gadolinium, CT venography, and conventional catheter angiography (gold standard) in case of slow blood flow, complex flow patterns, or parallel to imaging plane [21, 22, 25, 26]. Anyway, in pediatric population, less invasive techniques should be recommended and considered the first choice. Among MR methods, 3D PC MRV showed a robust overall quality with high-resolution images and low artifactual signal dropout. Furthermore, it appeared superior to 2D time-of-flight (ToF) MRV in depicting venous anatomy, resistant to saturation effects and less susceptible to artifacts. PC MRV can be comparable, and sometimes superior, to MRV with gadolinium. For this reason, it is considered the best technical approach to evaluate venous flow changes in posterior fossa also in other pathophysiologically similar diseases like idiopathic intracranial hypertension [27].

Using these techniques, the possible role of increased venous pressure first shown by Portnoy and Croissant [9] was progressively brought into evidence and associated with anatomical reductions of the sinus diameter at the level of the region of the torcular herophili [28, 29]. Assuming that arachnoid villi are hypoplasic or absent in infantile age [30–34], these authors proposed that increased venous outflow resistance could be responsible for impaired CSF resorption at the level of the capillary bed. Unfortunately, out of the six cases they described, 4 were affected by severe pre-existing conditions (3 meningitis, 1 severe prematurity with intraventricular hemorrhage) that affected significantly leptomeningeal integrity and CSF circulation, so they could not be considered as idiopathic external hydrocephalus [29]. The same significant bias was also present in all three cases they described in a subsequent paper [28]. The theory nevertheless gained further evidence in the report of Sainz et al., where significant restrictions of dural sinuses diameter were observed in 15 out of 17 patients diagnosed with idiopathic external hydrocephalus without significant associated pathologies. Moreover, the volume of subarachnoid spaces of the convexity correlated with the severity of the dural sinuses stenosis [16]. Unfortunately, the series was relatively small, and there was no control group.

The present report compares the anatomy of dural venous sinuses and the intracranial distribution of CSF volumes of a significant number of highly selected cases of idiopathic external hydrocephalus with a significant control group. Our data confirm that intracranial venous sinus anomalies have higher prevalence in idiopathic external hydrocephalus than commonly thought, especially in early infancy, also net of the technical limitations of MRV. Altered venous drainage, in fact, was detected in 84.53% of cases versus 25.33% of controls, and its presence was significantly related with the diagnosis of the widened subarachnoid spaces. Even considering the possible limits of the MRV technique, that offers only anatomical data without quantification of venous flow rate and/or speed, the high significance of these numbers remains striking. This association in fact exists in other conditions that share similar pathophysiological features in this age range like achondroplasia and syndromic craniosynostosis. In these diseases, the functional cascade: anatomical stenosis/obstruction of dural sinuses > consequent increased venous outflow resistance > increased venous pressure has been already demonstrated both clinically and experimentally as the underlying cause of increased CSF outflow resistance ultimately leading to macrocrania, SAS dilatation, increased intracranial pressure, and possibly hydrocephalus depending upon several factors like the severity of sinus obstruction, age of the child, and state of the cranial vault sutures [18]. The hypothesis that the pathophysiology of external hydrocephalus could share the same underlying mechanism was therefore intuitive, and our data simply support the original theory of Portnoy and Croissant [9] and the preliminary MRV observation [16, 28]. Further studies will necessarily include functional evaluation of venous flow rate and speed.

The anatomical severity of the venous alterations appeared to be also significantly related to the volume of SAS, as already reported by Sainz [16], although in our study, this association was not very strong. This can be related with the limitations and difficulty of MRV technique in the evaluation of amount and effectiveness of the collateral circulation network. Moreover, the number of variables to consider is relatively high: the development of venous sinus anatomy in the neonatal period is a very dynamic and complex process, prompted by the shift from fetal to neonatal circulation and favored by the progressive postural change [35]. This process leads from a diffuse and irregular intradural venous network in the infratentorial space to the constitution of the well-known inverted “T” anatomy of the sagittal sinus dividing into the two transverse sinuses. This process takes place in the earliest weeks of life [36].

The significantly higher incidence of postural plagiocephaly in patients with external hydrocephalus compared with the control group is in line with other reports of the literature describing series of patients associating macrocrania and postural plagiocephaly [37, 38]. These authors proposed the possible link between increased intracranial fluid and increased malleability of the calvaria finally leading to positional molding. In fact, further studies demonstrated that this association may possibly play a role only for the macrocranic patients with positional plagiocephaly, because patients with normal head circumference and positional plagiocephaly do not show any difference in intracranial fluid content at a more accurate measurement of intracranial fluid volume [39]. Our series shows that although positional plagiocephaly is very frequent in patients with external hydrocephalus, the majority of these patients present a regular shape of the head without any positional molding. This supports the idea that increased intracranial fluid content is unlikely to be the main etiological factor for positional plagiocephaly but, instead, a possible consequence of it.

The significant association between the highest degree of venous obstruction and the flattened side of plagiocephaly shown in this study suggests the possibility that the significant calvarial deformation observed in positional plagiocephaly could impair the formation or development of the transverse-sigmoidal axis on the same side. Some authors observed in adults an increased venous outflow through the dural sinuses in the passage from a supine position to a prone position [15]. The transverse sinuses, which increase rapidly in size during fetal life from the 17th through 30th week of gestational age assuming a ballooning appearance, progressively decreases its caliber after birth and reaching its adult shape and caliber around the age of 1 year, with only minor increases between 1 and 6 years of age [35]. The asymmetric flattening of the occipital calvaria observed in positional plagiocephaly sometimes is already visible at birth and could interfere with the dynamic process of formation of the transverse sinus of the same side, determining preferential venous flow through the uncompressed side, finally resulting in hypoplasia, stenosis, or agenesia of the transverse sinus on the flattened side. It can be hypothesized that the supine position that should be maintained in children in order to prevent the sudden infant death syndrome [1] could determine, in case of existing positional plagiocephaly, slower venous outflow through an already compressed dural sinus. This flow reduction could be responsible of progressive narrowing of an already hypoplasic sinus, finally leading to obstruction or agenesis. These anatomical and functional variations could determine additional obstacle to venous outflow, being finally responsible for further increase of venous outflow resistance, impaired CSF resorption, and widening of pericerebral spaces in our population of patients with macrocrania. The same mechanism could be hypothesized to explain the widening of subarachnoid spaces of the convexity observed in patients with positional plagiocephaly without macrocrania.

This study has several limitations: the control group is not formed, by definition, by “healthy volunteers” because MRI in this age range is performed under general sedation and is performed only for significant clinical concerns; for these reasons, the control group is smaller than the case group and has different gender ratio; MRV technique used offers a purely anatomical figure of major vessels without quantitative data of blood flow, and probably inadequate study of collateral venous system; the intracranial CSF volumes studied includes only the pericerebral and the ventricular spaces above the intercommissural plane; not all patients could be adequately tested under the developmental point of view because clinical observation was performed over a very long period of time. In spite of these limitations, the high number of patients studied by MRV, the high number of venous anomalies observed, and the significant association between occipital flattening and anatomical venous obstruction on the same side offer new insights on the pathophysiology of this condition and warrant further directions of investigation.

## Conclusions

The high rate of abnormalities of venous dural sinuses anatomy observed in a significant number of patients followed up for external hydrocephalus offers significant anatomical data to support the hypothesis of increased venous outflow resistance and consequent intracranial venous hypertension in the pathophysiology of this condition. The significantly higher incidence and severity of venous anomalies on the flattened side of plagiocephalic patients offers new insights and warrants further studies on the possible impact of calvarial flattening on the ipsilateral dural sinus patency.

## Supplementary information

Below is the link to the electronic supplementary material.

Supplementary file1 (PDF 83328 KB)

## Data Availability

Yes.
