# Supplementary material for: Dural venous sinus anatomy in children with external hydrocephalus: analysis of a series of 97 patients
Source: Childs Nerv Syst. 2021 Aug 24;37(10):3021–32. doi: 10.1007/s00381-021-05322-5 (PMC8510989; doi:10.1007/s00381-021-05322-5)

DURAL VENOUS SINUS ANATOMY IN CHILDREN WITH EXTERNAL HYDROCEPHALUS. ANALYSIS OF A SERIES OF 97 PATIENTS.

Giuseppe Cinalli[1] MD, IFAANS, FACS, Giuliana di Martino[1] MD, Carmela Russo[2] MD, Federica Mazio[2] MD, Anna Nastro[2] MD, Giuseppe Mirone[1] MD, Claudio Ruggiero[1] MD, Ferdinando Aliberti[1], MD, Daniele Cascone[2] MD, Eugenio Covelli[2] MD and Pietro Spennato[1] MD

Department of Pediatric Neurosciences, Pediatric Neurosurgery Unit, Santobono-Pausilipon Children's Hospital, Naples, Italy
Department of Pediatric Neurosciences, Pediatric Neuroradiology Unit, Santobono-Pausilipon Children's Hospital, Naples, Italy

SUPPLEMENTARY MATERIAL

VENOUS OBSTRUCTION GRADING SCORE (VOGS)

RADIOLOGICAL VENOUS MAGNETIC RESONANCE (MRV) GRADING OF DURAL VENOUS SINUS STENOSIS/OCCLUSION

Radiological grading of patterns of anatomical variations of dural venous sinuses in the region of Torcular Herophili and posterior cranial fossa.

VENOUS OBSTRUCTION GRADING SCORE (Cinalli G et al)

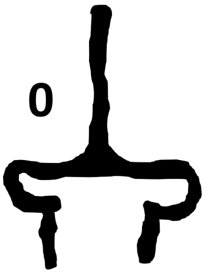

Grade 0: Normal Sinus anatomy

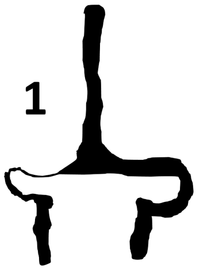

Grade 1: Unilateral stenosis

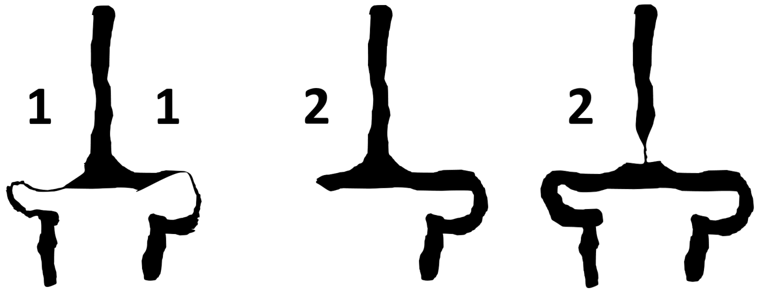

Grade 2A (left): Bilateral stenosis
Grade 2B (center): Unilateral gap
Grade 2C (right): Sagittal sinus stenosis

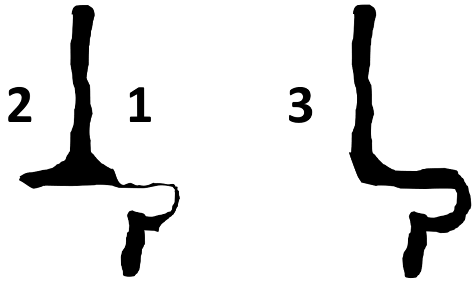

Grade 3A (left): Gap + contralateral stenosis
Grade 3B (right): Unilateral aplasia

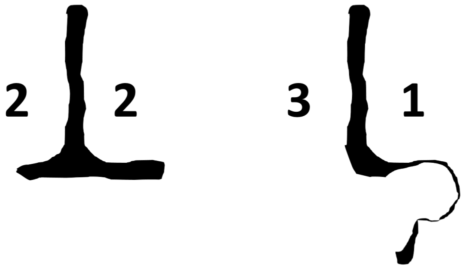

Grade 4A (left): Bilateral gap
Grade 4B (right): Aplasia + contralateral stenosis

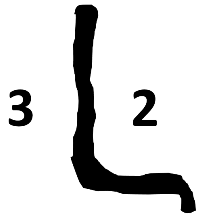

Grade 5: Aplasia + contralateral gap

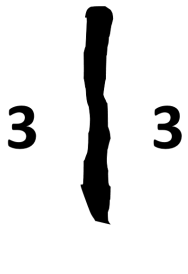

Grade 6: Bilateral aplasia

<u>Supplementary Figure 1: Grade 0</u>: Four examples of normal anatomy of dural venous sinuses at the level of Torcular Herophili, posterior fossa and jugular foramen. Transverse sinuses and sigmoid sinus are roughly symmetric without significant variations of diameter and no images of flow gap.

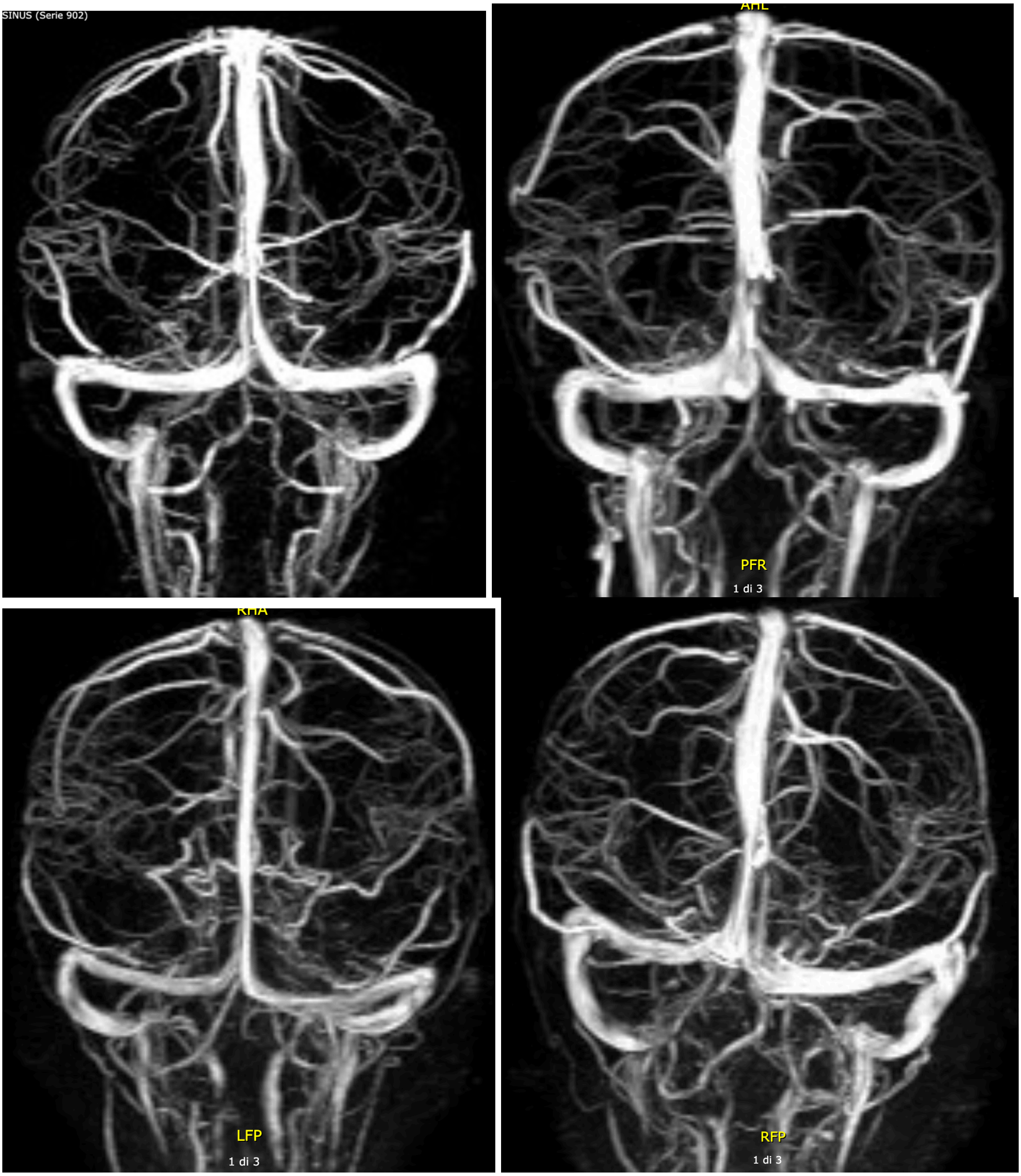

<u>Supplementary Figure 2: Grade 1</u>: Unilateral stenosis of the transverse sinus (red arrowhead) on the right side (A-C-D) and on the left side (B).

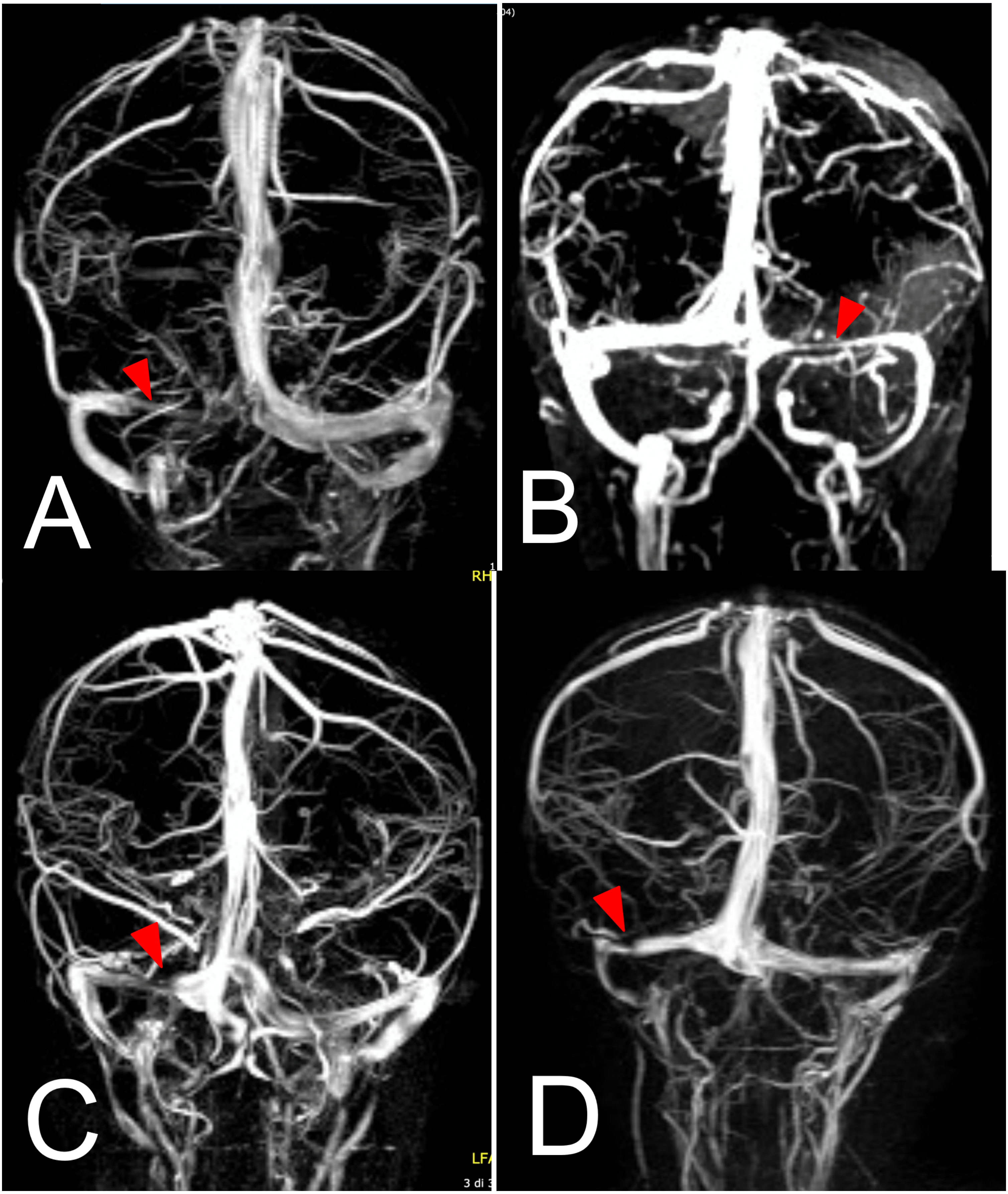

<u>Supplementary Figure 3</u>: **<u>Grade 2A</u>**: Bilateral stenosis (arrows) of the middle third of both transverse sinuses (A-B-C) and of the lower third of both sigmoid sinuses (D).

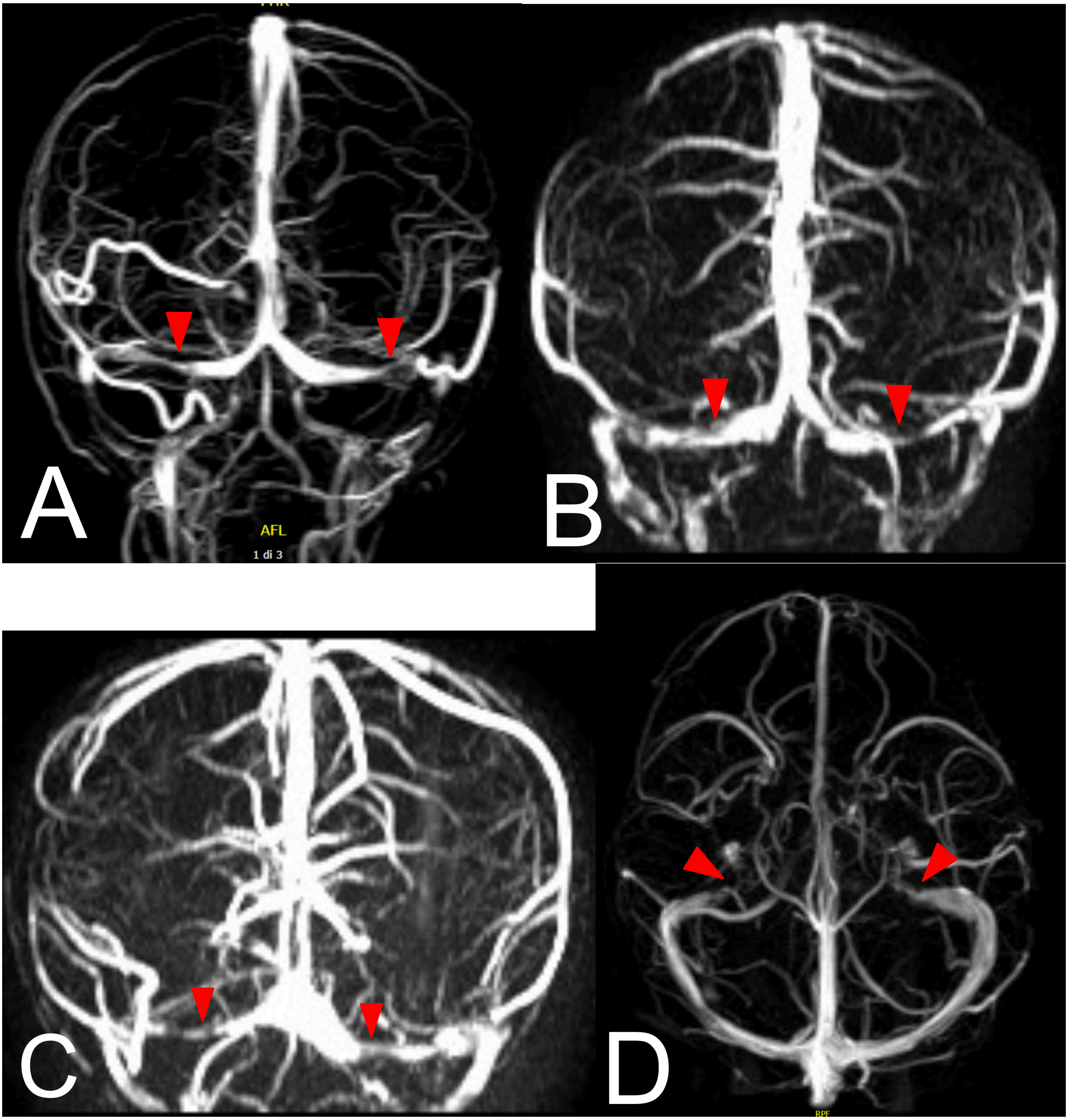

Supplementary Figure 4: Grade 2B: Unilateral flow gap o the proximal (A-B) and of the middle third (C-D) of the right transverse sinus (double red arrowheads).

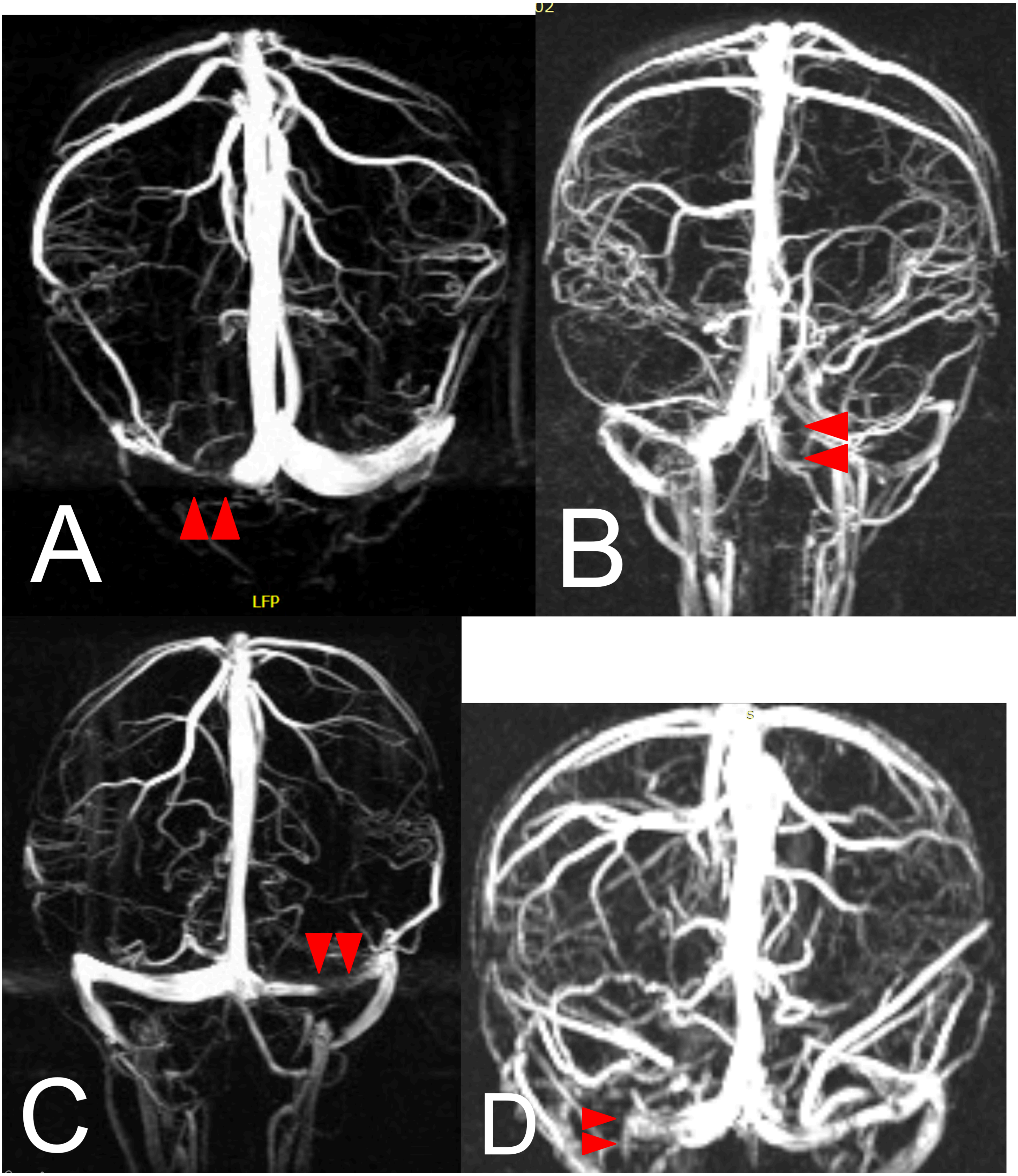

<u>Supplementary Figure 5: Grade 2C</u>: Stenosis of the terminal part of the superior sagittal sinus (double red arrowheads). The stenosis is well visible both in coronal (A) and sagittal views (B) and is confirmed in standard axial T2 images (C) where the sinus diameter is significantly smaller if compared to upper cuts (D).

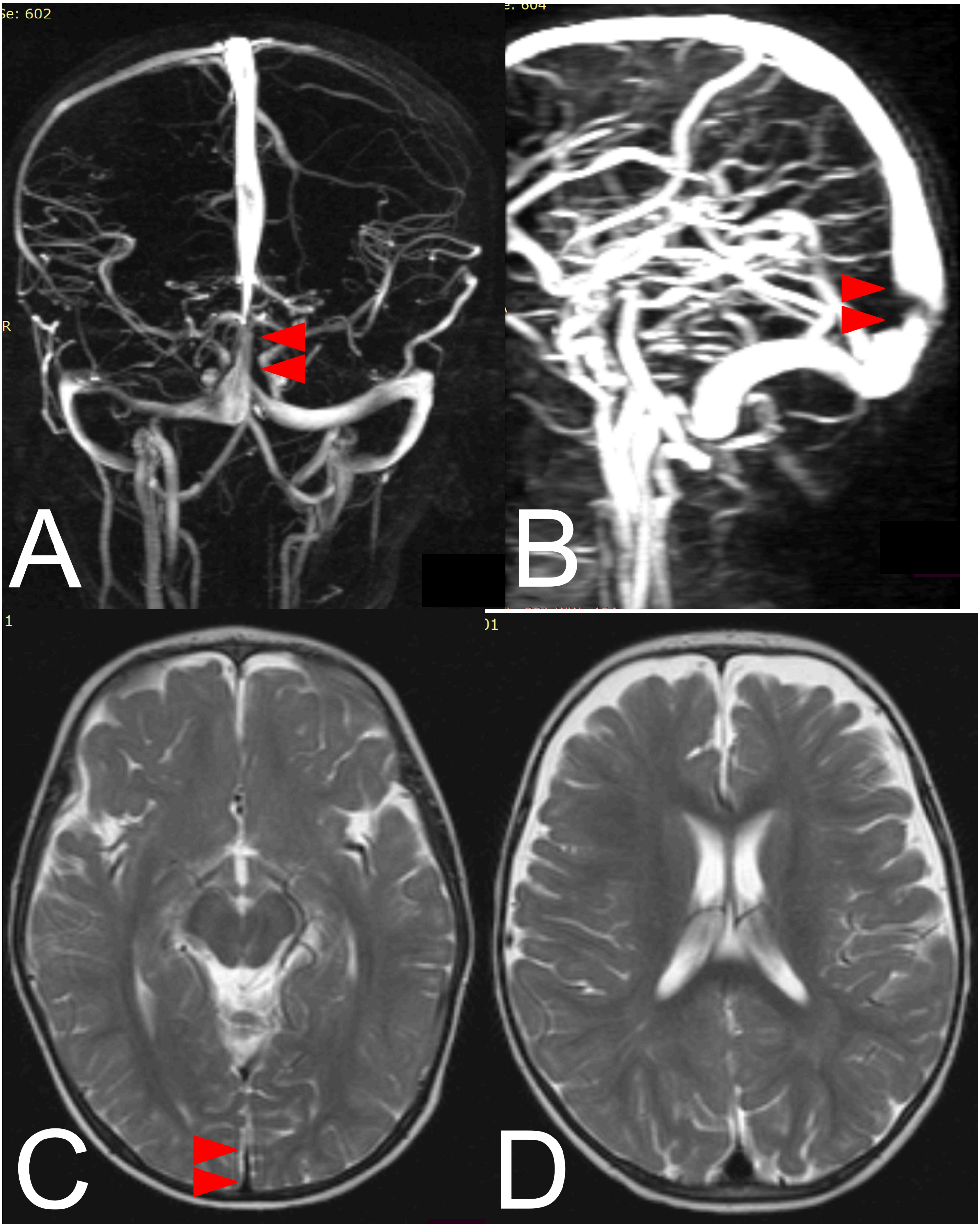

Supplementary Figure 6: **Grade 3A**: Flow gap of the right transverse sinus (double arrowheads) and stenosis of the left (arrowhead) transverse sinus (A-B). Flow gap of the left transverse sinus (double arrowheads) and stenosis of the right transverse sinus (arrowhead) (C-D).

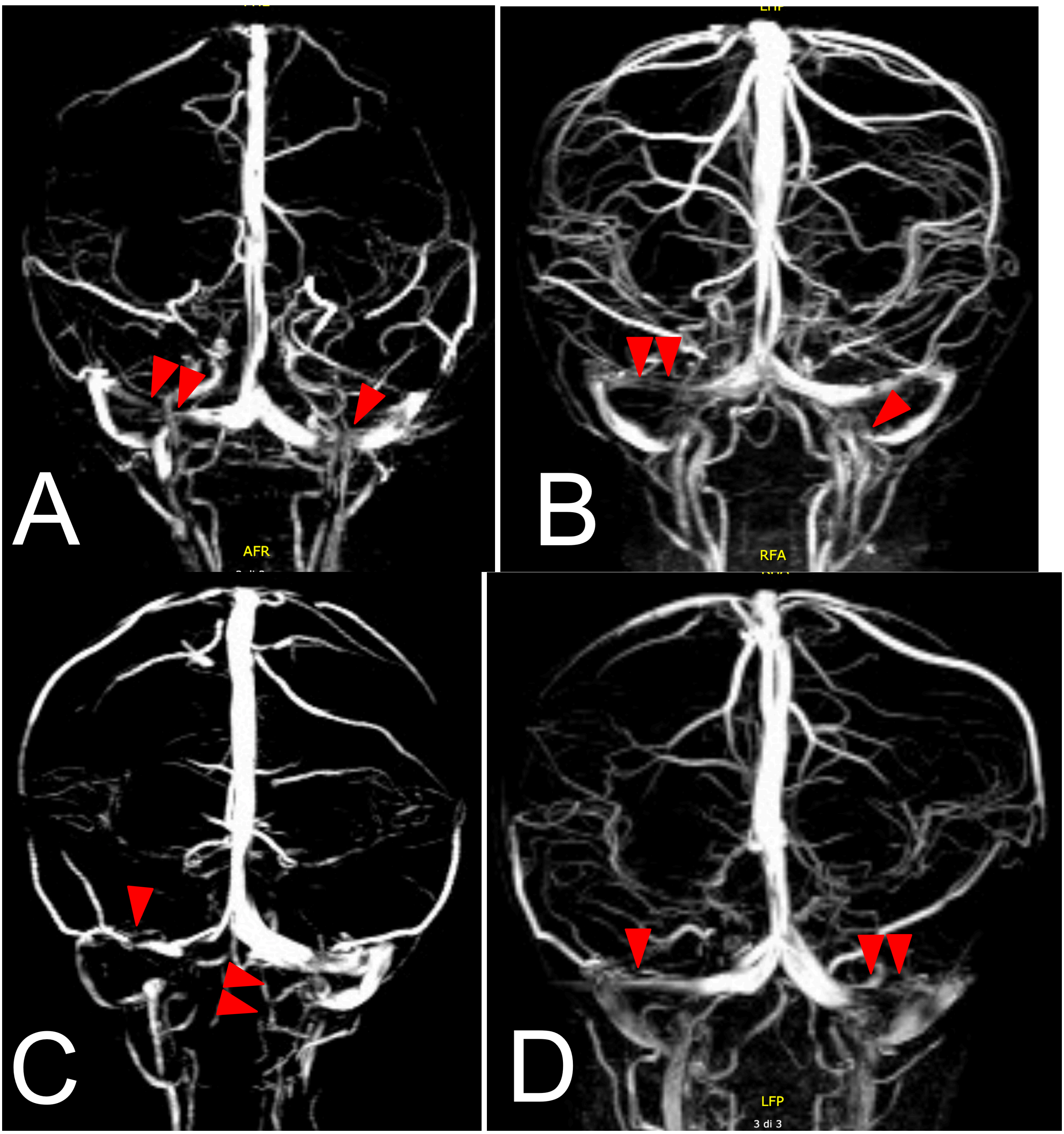

Supplementary Figure 7: Grade 3B: Agenesis of the right (A-D) and of the left (B-C) transverse sinus (triple red arrowheads).

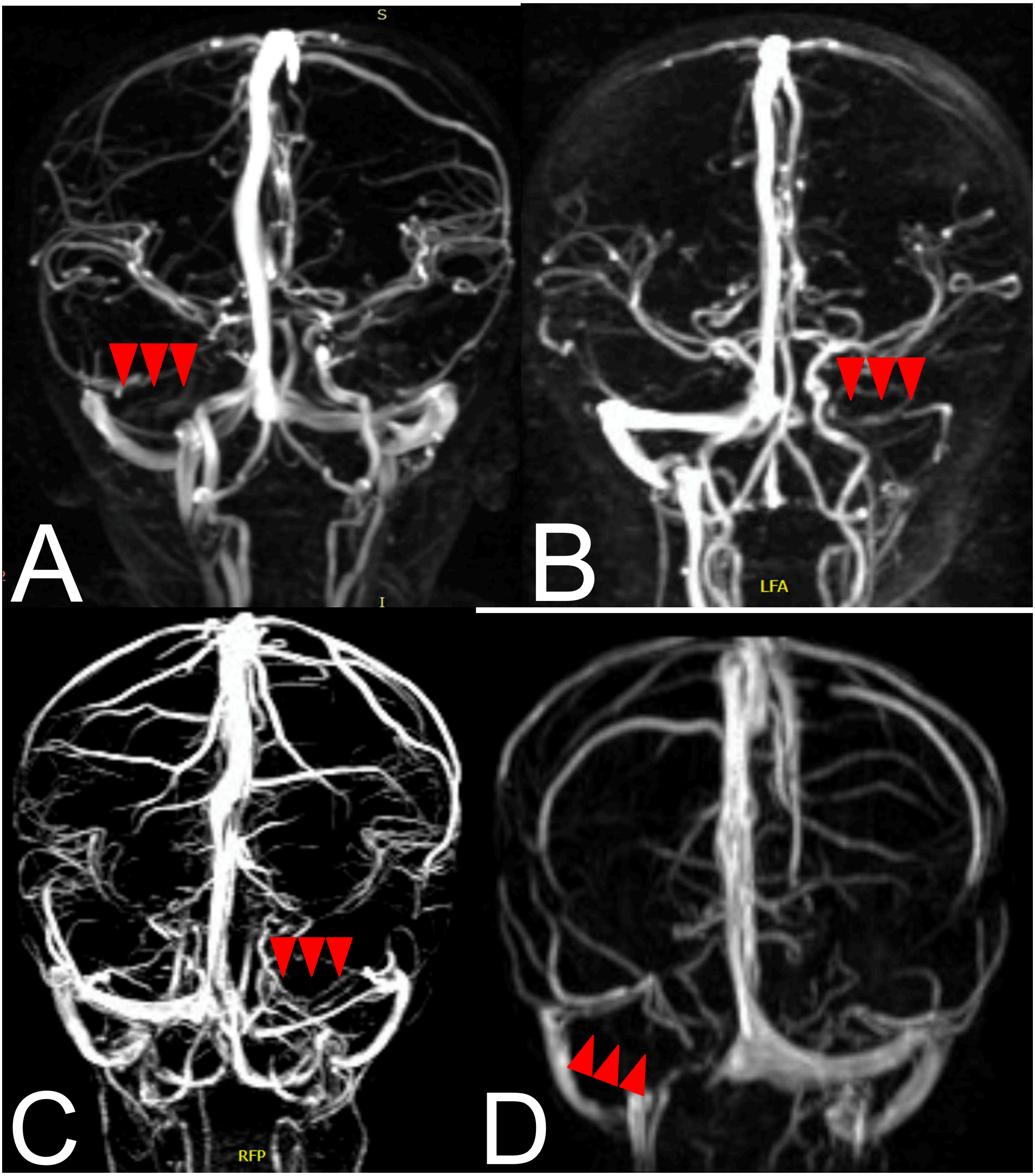

Supplementary figure 8: Grade 4A: Bilateral flow gap of both transverse sinuses. (A-B-C-D)

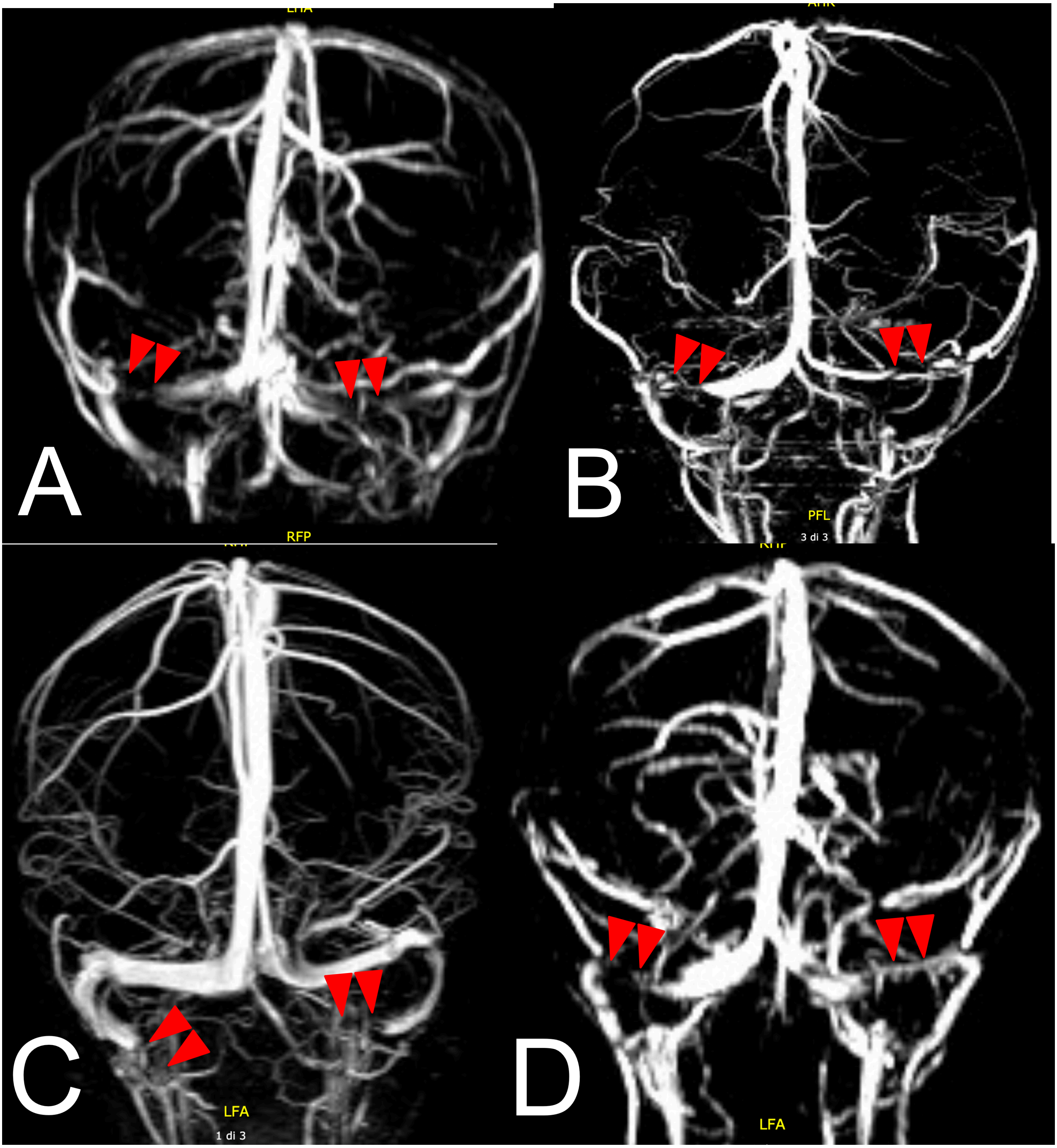

Supplementary figure 9: Grade 4B: Agenesis (A-C) of the right transverse sinus (triple arrowheads) and stenosis of the left transverse sinus (arrowhead). Agenesis (B-D) of the left transverse sinus (triple arrowheads) and stenosis of the right transverse sinus (arrowhead).

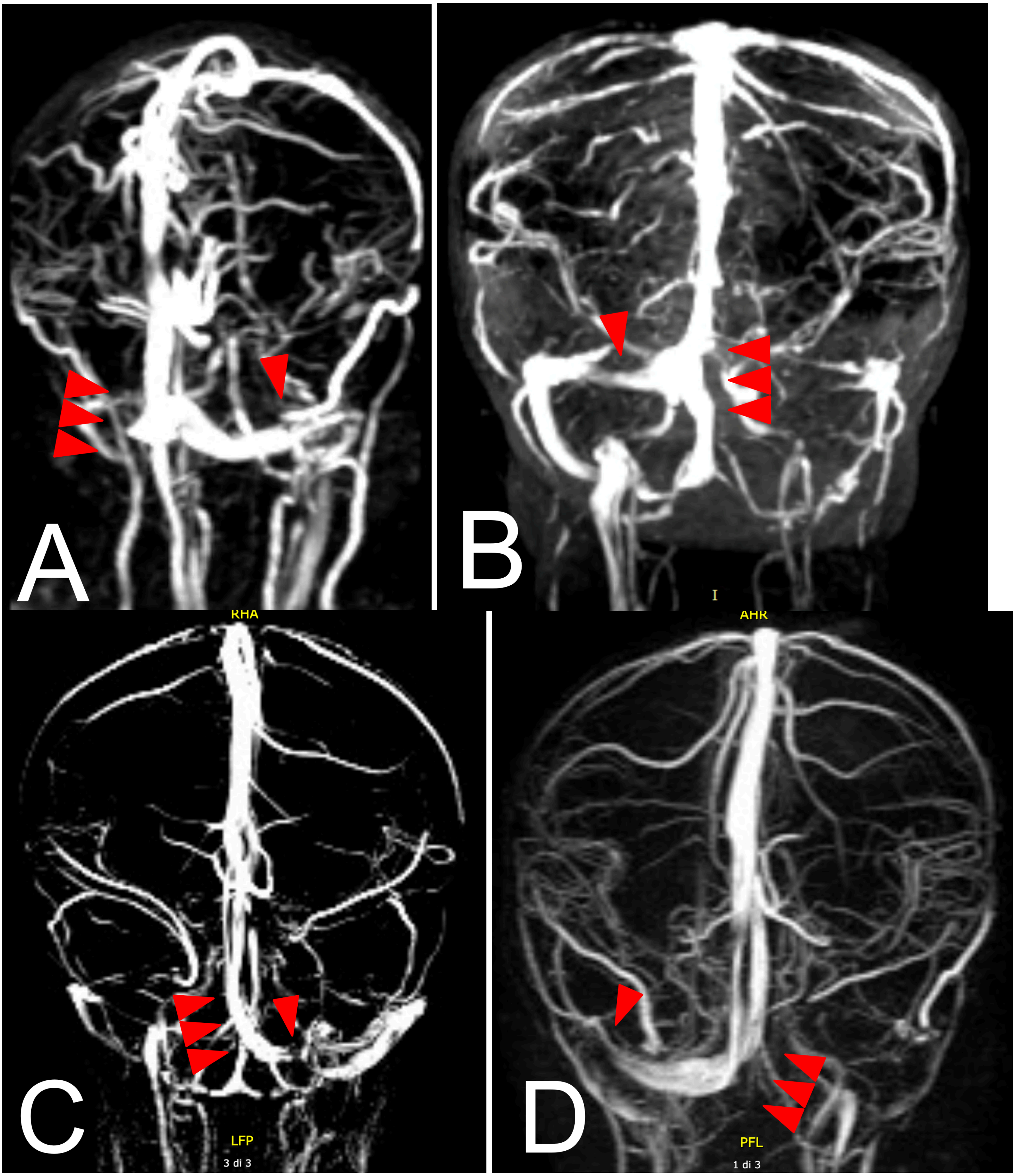

<u>Supplementary figure 10:</u> **<u>Grade 5</u>**: Agenesis of the right transverse sinus (3 arrowheads) and flow gap of the left sigmoid sinus (2 arrowheads) (A-C). Agenesis of the left transverse sinus (3 arrowheads) and flow gap of the right sigmoid sinus (2 arrowheads) (B-D).

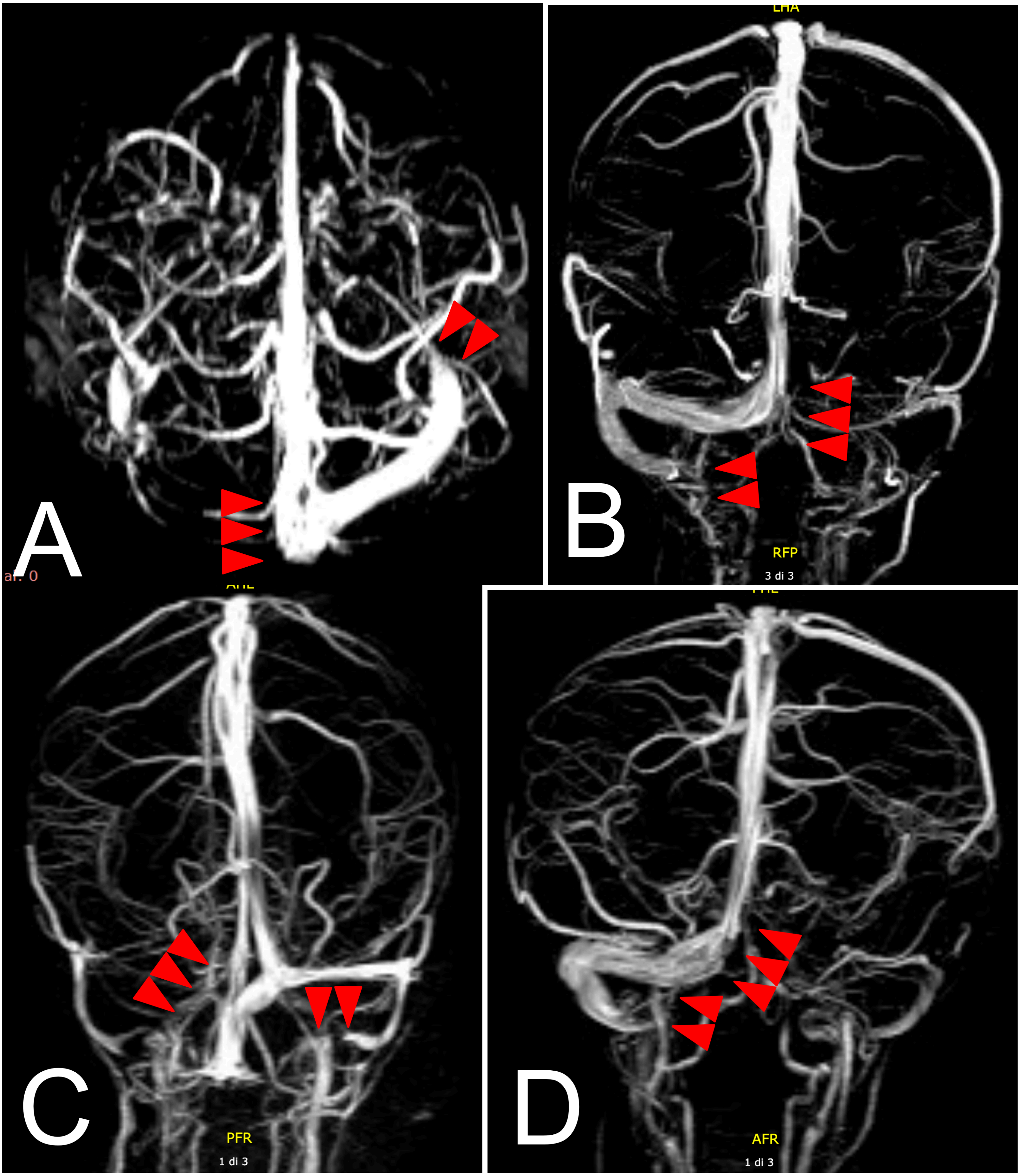

Supplementary figure 11: Grade 6: Bilateral agenesis of both transverse sinuses visible in coronal (A) and axial (B) view.

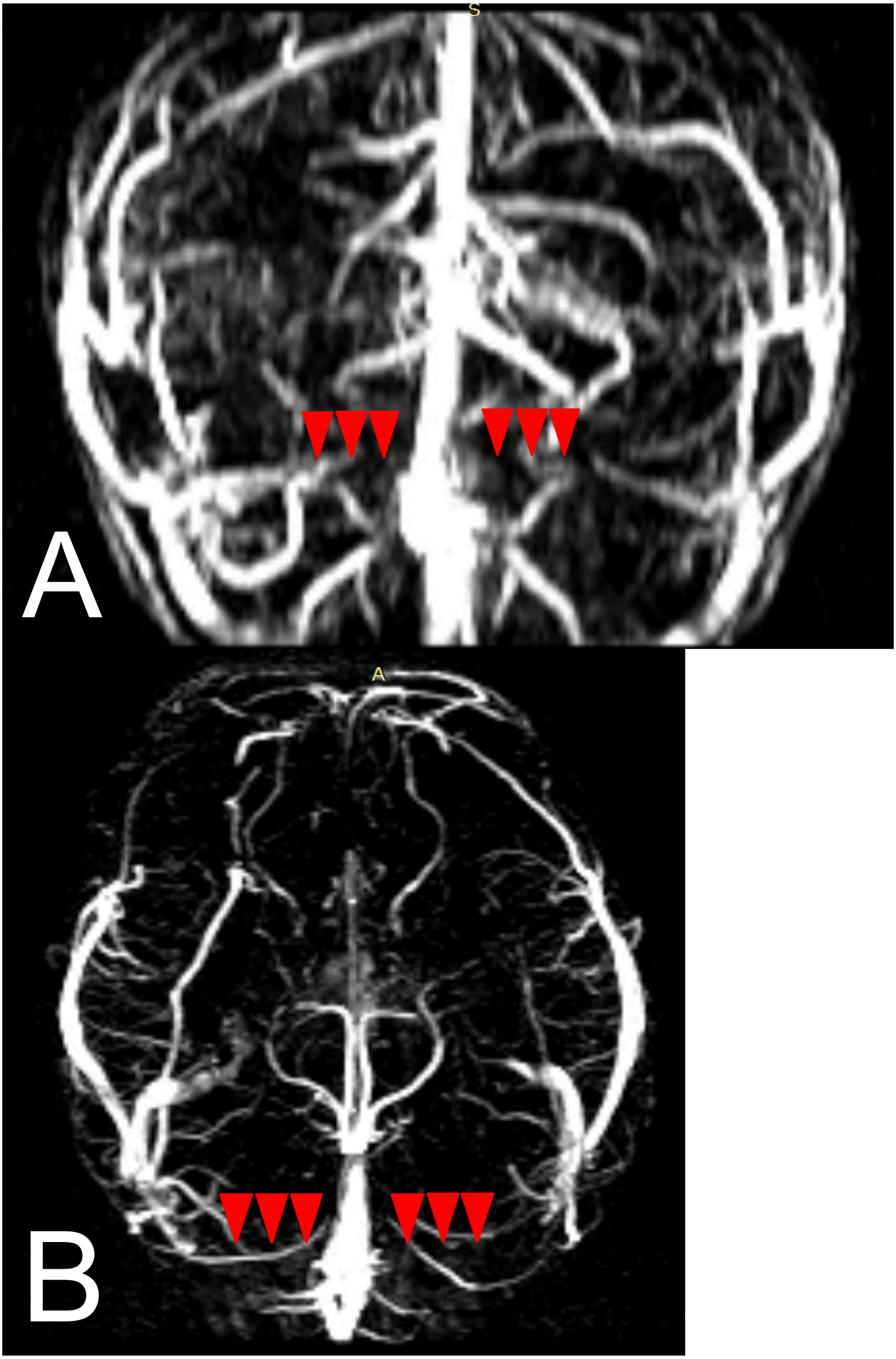

Supplement: Supplementary file 1 — Supplementary file1 (PDF 83328 KB) [file 381_2021_5322_MOESM1_ESM.pdf]
